# FTO downregulation-mediated m6A modification resulting in enhanced hepatocellular carcinoma invasion

**DOI:** 10.1186/s13578-025-01395-w

**Published:** 2025-05-02

**Authors:** Cheng Zhou, Yong Zhang, Shi-Ming Shi, Dan Yin, Xue-Dong Li, Ying-Hong Shi, Jian Zhou, Zheng Wang, Qing Chen

**Affiliations:** 1 Department of Liver Surgery and Transplantation, Liver Cancer Institute, Zhongshan Hospital, Key Laboratory of Carcinogenesis and Cancer Invasion of Ministry of Education, Fudan University, Shanghai, 200032 China; 2 Department of General Surgery, Zhongshan Hospital, Fudan University, Shanghai, 200032 China; 3 Department of Vascular, Thyroid, and Breast Surgery, Affiliated Hospital of Guangdong Medical University, Zhanjiang, Guangdong Province 524000 China; 4 Institute of Biomedical Sciences, Fudan University, Shanghai, 200032 China; 5 State Key Laboratory of Genetic Engineering and Collaborative Innovation Center for Genetics and Development, School of Life Sciences, Fudan University, Shanghai, 200032 China; 6 Department of Liver Oncology, Zhongshan Hospital (Minhang Meilong), Fudan University (Shanghai Geriatric Medical Center), Shanghai, 201104 China

**Keywords:** Hepatocellular carcinoma, Fat mass and obesity-associated protein, N6-methyladenosine, VEGFA

## Abstract

**Background:**

Dysregulation of N6-methyladenosine (m6A) modifications has been implicated in various cancers, including hepatocellular carcinoma (HCC). This study aimed to elucidate the role of m6A modifications in HCC prognosis and the molecular mechanisms involved, particularly focusing on the demethylase FTO.

**Methods:**

We analyzed m6A expression in a cohort of 323 HCC patients using immunohistochemical (IHC) staining. The expression of m6A-related genes (FTO, ALKBH5, METTL3, METTL14) was evaluated by qRT-PCR in 120 paired HCC tissues. Further, we established HCC cell lines with altered FTO expression to assess its impact on cell proliferation, invasion, and metastasis through various in vitro assays and in vivo orthotopic HCC mouse models. Statistical analyses included Pearson chi-square test, Kaplan-Meier survival analysis, and both univariate and multivariate Cox regression analyses.

**Results:**

IHC staining revealed elevated m6A levels in HCC tissues compared to adjacent non-tumorous tissues, with 57.3% of HCC patients showing increased m6A expression. High m6A levels were correlated with poorer overall survival (OS) and recurrence-free survival (RFS) rates. FTO, a demethylase, was significantly downregulated in HCC tissues and cell lines, particularly in highly metastatic lines. Overexpression of FTO in HCC cells reduced proliferation, migration, and invasion, whereas FTO knockdown had the opposite effect. In vivo, FTO overexpression decreased tumor growth and metastasis. RNA-Seq analysis identified VEGFA as a key gene downregulated by FTO, implicating its role in angiogenesis and tumor progression.

**Conclusions:**

Our findings suggest that elevated m6A levels are associated with poor prognosis in HCC patients. FTO downregulation contributes to aberrant m6A modifications, promoting HCC progression and metastasis. FTO acts as a tumor suppressor by negatively regulating VEGFA expression, highlighting its potential as a therapeutic target for HCC treatment. These results highlight the significance of m6A modifications in HCC and provide a foundation for future research on targeted therapies.

**Supplementary Information:**

The online version contains supplementary material available at 10.1186/s13578-025-01395-w.

## Introduction

Hepatocellular carcinoma (HCC), representing approximately 75–85% of primary liver cancers, is a prevalent malignancy and ranks as the third leading cause of cancer-related mortality worldwide in 2020 [1]. An estimated 841,000 new cases and 782,000 deaths occurred annually globally. The incidence rates and risk factors for HCC exhibit variations across diverse geographical regions. Nevertheless, on a global scale, obesity and type 2 diabetes have emerged as significant etiological factors in HCC [2]. The increasing global prevalence of obesity and type 2 diabetes is considered a contributing factor to the observed rising incidence of HCC [3].

The fat mass and obesity-associated protein (FTO) have been substantiated to have pivotal associations with obesity and m6A methylation in previous studies [4–6]. N6-methyladenosine (m6A) is the most abundant modification in mRNA and is involved in nearly all stages of the RNA lifecycle, including transcription, maturation, translation, degradation, and stability [7]. It plays a crucial regulatory role in cellular differentiation, proliferation, and aging and is involved in the regulation of physiological and pathological processes, including cancers [8]. The m6A methylation enzymes consist of three types: m6A “writers”, m6A “erasers”, and m6A “readers”. The m6A readers, also known as m6A recognition proteins, selectively recognize the m6A modification of target RNA and participate in various stages of RNA metabolism, including proteins from the YTH family, hnRNP superfamily proteins, EIF3A, and various other proteins [9]. The m6A writers refer to the m6A methyltransferase complex, which catalyzes the transfer of a methyl group from S-adenosyl methionine to the N6 position of adenine. The m6A writers include METTL3, METTL14, WTAP, KIAA1429, RBM15, HAKAI, ZC3H13, and METTL16 [10]. The m6A erasers, known as m6A demethylases, erase the m6A methylation modification of the target RNA. Currently, only two m6A demethylases have been discovered: FTO and alkB homolog 5 (ALKBH5), both of which independently affect the erasure of m6A modifications in RNA [10]. Among all m6A-associated genes, FTO is renowned for promoting obesity [11], which is an important driving factor for HCC. One fourth of the world’s population is thought to suffer from nonalcoholic fatty liver disease (NAFLD). The incidence of nonalcoholic steatohepatitis is predicted to rise by as much as 56% in the next decade. NAFLD is the most rapidly expanding cause of HCC in the United States, France, and the United Kingdom [12]. Internationally, the prevalence of NAFLD-related HCC is expected to increase in tandem with the escalating obesity crisis. The estimated annual occurrence of HCC varies from 0.5 to 2.6% among NASH cirrhosis patients [12]. Urgent measures that enhance global awareness and address metabolic risk factors are imperative to mitigate the impending burden of NAFLD-related HCC. FTO, which is regarded as the gene with the strongest association with obesity that has been discovered thus far, could potentially be a determinant of NAFLD-associated HCC.

Prior research has established a strong correlation between FTO and the advancement of HCC. Removal of FTO restrains the proliferation and expansion of tumors within the body, leading to G0/G1 phase arrest. FTO expedites the generation of translated products by instigating the demethylation of PKM2 mRNA [13]. The phosphorylation and ubiquitination levels of FTO are influenced by AMD1 and IQGAP1, thereby altering the biological characteristics of HCC [14]. SIRT1 has the ability to disrupt the stability of FTO and regulate the downstream molecular m6A modification in the development of HCC tumors, thereby promoting the invasion and metastasis of HCC [15]. Non-coding RNA, such as circGPR137B, can elevate the expression of FTO through the circGPR337B/miR-4739/FTO feedback loop, consequently inhibiting the development and metastasis of HCC tumors [16]. Despite the confirmed role of FTO in liver cancers, there is currently an absence of research on the m6A regulation mediated by FTO.

In the present study, we delved into and substantiated the correlation between m6A, FTO, and HCC. We corroborated the influence of FTO on the invasive and metastatic characteristics of HCC cells and authenticated the mechanisms by which FTO suppresses the invasive and metastatic capacities of HCC.

## Materials and methods

### Patients and follow-up

The study obtained ethical approval from the Institutional Ethics Committee of Zhongshan Hospital (B2022-216R) and acquired informed consent from each patient. Two distinct cohorts of 443 HCC patients were included in this study. The 120 tumor tissues and paired adjacent nontumor liver tissues utilized in RT-qPCR analysis were sequentially gathered from patients who underwent curative resection between January and December 2010 (cohort 1, *n* = 120, snap-frozen tissues). Paraffin-embedded tissues from cohort 2 were randomly obtained from HCC patients who underwent curative resection from 2003 to 2004 at the Liver Cancer Institute, Zhongshan Hospital, Fudan University (cohort 2, *n* = 323). Patients in cohort 1 were monitored after surgery until March 15, 2013, whereas patients in cohort 2 were monitored until March 15, 2009.

The histopathological diagnosis was established in accordance with the criteria set forth by the World Health Organization. The histological grade of tumor differentiation was ascertained using the classification outlined by Edmondson and Steiner [17]. Liver function was evaluated using the Child-Pugh scoring system. Tumor stage was determined based on the 2010 International Union Against Cancer tumor-node-metastasis classification system. Postsurgical patient surveillance was conducted in line with previously established protocols [18, 19]. The overall survival (OS) was defined as the duration between the surgical procedure and either decease or the final observation point. In the case of surviving patients, the data were terminated at the most recent follow-up. The time to recurrence (TTR) was defined as the duration between the surgical date and the identification of any recurrence (intrahepatic relapse and extrahepatic metastasis).

### Cell lines and animals

The study utilized four human HCC cell lines demonstrating varying metastatic potentials (HepG2, MHCC97L, MHCC97H, and HCCLM3), along with a human non-transformed hepatic cell line L-02. The HepG2 cell line was acquired from the Cell Bank of the Chinese Academy of Sciences in Shanghai, China. Additionally, HCC cell lines with progressive metastatic potential (MHCC97L, MHCC97H, and HCCLM3, sharing the same genetic background but differing in metastatic potentials) were established at our institute [20], and validated through short tandem repeat analysis during the study period. The cells were consistently cultured in DMEM supplemented with 10% FBS, 100 U/mL penicillin, and 100 mg/mL streptomycin at 37℃ with 95% air and 5% CO_2_.

Male BALB/c nude mice (4 weeks old) were procured from the Shanghai Institute of Material Medicine, Chinese Academy of Science, and were raised under specific pathogen-free conditions. All procedures were conducted in compliance with the Guideline for ethical review of animal welfare in China (GB/T 35892 − 2018).

### RNA isolation and qRT-PCR

RNA was extracted from the cells and frozen tumor specimens using Trizol reagent (Invitrogen, Carlsbad, CA, USA), and cDNA synthesis was carried out using a high capacity of cDNA Reverse Transcription kit (Applied Biosystems, Life Technologies, Carlsbad, CA, USA) following the manufacturer’s protocol. The quantitative real-time polymerase chain reaction (qRT-PCR) was performed as previously described [16], and the primers utilized for qRT-PCR were detailed in Supplementary Table S1.

### Western blot analysis

The protein concentrations of cellular or nuclear extracts were ascertained using a bicinchoninic acid Assay Kit (Bio-Rad). Essentially, equivalent quantities of protein lysate fractions were separated through SDS-PAGE, transferred onto PVDF membrane, and immunoblotted as detailed in our previous study [19]. The antibodies employed are specified in Supplementary Table S2.

### RNA sequencing

The extraction of total RNA was conducted using the ESscience RNA-Quick Purification Kit (YiShan Biotech, Shanghai, China). Subsequently, library construction and RNA-sequencing (RNA-seq) were carried out at Shanghai Biomedical Lab Co Ltd (Shanghai, China) utilizing the Illumina NovaSeq 6000 (Illumina, USA). The raw data underwent normalization, and clean reads were obtained by removing adapter and poly-N sequences, as well as low-quality reads. Differential expression analysis was performed using the edgeR package in R software (v.4.1.2). Moreover, the computational analysis provided by the same entity was employed. To identify differentially expressed genes, a threshold of a *P* value < 0.01 and a fold change > 2 or < 0.5 were set as the criteria.

### In vivo assays for tumor growth and metastasis

Animal experiments were conducted with the approval of the Ethics Committee of the Zhongshan Hospital Biomedical Research Department. All surgical procedures and animal care complied with the Guideline for ethical review of animal welfare in China (GB/T 35892 − 2018). Briefly, cells (5 × 10^6^) were suspended in 100 µl serum-free DMED and injected subcutaneously into the upper left flank region of nude mice. For the liver orthotopic transplantation models, standardized procedures described previously were followed [21]. At the end of the study, mice were euthanized, and tumor tissues were collected, photographed, and the tumor volume was calculated in mm^3^ using the formula: V = *ab*^*2*^/2, where a and b represented the largest and smallest tumor diameters measured at necropsy, respectively [22]. The Lungs were dissected, embedded in paraffin, and the total number of lung metastases was counted under a microscope. The metastases were categorized into four grades based on the number of tumor cells present at the maximal section for each metastatic lesion: grade I (≤ 20 tumor cells), grade II (20–50 tumor cells), grade III (50–100 tumor cells), and grade IV (> 100 tumor cells).

### Statistical analysis

The clinical and pathological characteristics were presented as frequencies and percentages for categorical covariates, and medians and ranges for continuous covariates. Comparison of continuous variables was performed with Student’s *t*-test or Mann-Whitney *U* test, as appropriate, while categorical variables were assessed using the *χ2* test or Fisher’s exact test. Colorful-clouds The OS and RFS were assessed using the Kaplan-Meier method, with differences analyzed by the log-rank test. Univariate and multivariate analyses were conducted using the Cox proportional hazards regression model. Statistical analyses were carried out using SPSS 26.0 software (IBM, New York, USA). A two-tailed *P* value of less than 0.05 was considered statistically significant for all analyses.

## Results

### Abnormal m6A modifications in HCC correlates with poorer prognosis

**Fig. 1:**
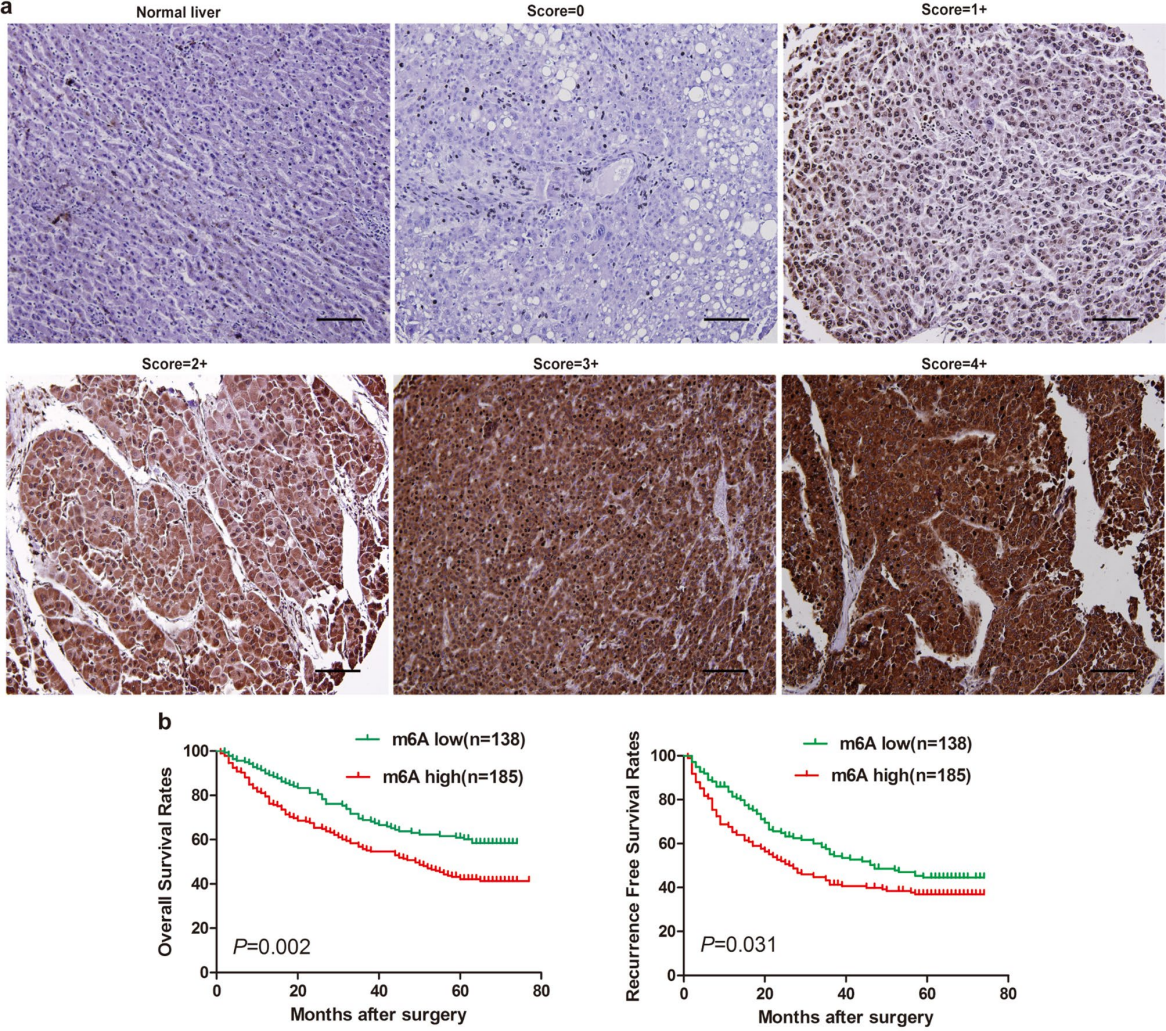
Abnormal m6A modifications in HCC correlates with poorer prognosis. (**a**) Based on the intensity of m6A expression in the nucleus and cytoplasm in HCC tissue microarray (cohort 2, *n* = 323) as determined by immunohistochemical staining, it was categorized into score 0–4+, where score 0, 1+, and 2 + were defined as the low expression group, and score 3 + and 4 + were defined as the high expression group. (**b**) Patients with high m6A expression in tumor cells exhibited reduced overall survival and recurrence-free survival (cohort 2, *n* = 323). Group differences were assessed using the Kaplan-Meier method. HCC, hepatocellular carcinoma

To investigate the potential impact of m6A on the clinical prognosis of patients with HCC, we analyzed its expression in a cohort of 323 individuals diagnosed with HCC (cohort 2, *n* = 323). Employing m6A immunohistochemical (IHC) staining, we observed robust staining in the nuclei and cytoplasm of malignant cells within HCC tissues, while the majority of adjacent liver tissues showed either partial or complete loss of m6A. Notably, the immunohistochemistry results unequivocally demonstrated predominant localization of m6A within the nucleus and cytoplasm (Fig. 1a). Furthermore, a considerable number of tumor tissues exhibited significantly elevated levels of m6A compared to the corresponding adjacent non-tumorous tissues, with 185 out of 323 patients (57.3%) demonstrating increased m6A expression. The Pearson chi-square test revealed a robust correlation between m6A expression and key clinical parameters such as age (*P* = 0.017), microvascular invasion (*P* = 0.013), and tumor differentiation (*P* = 0.038, Supplementary Table S3).

By the last follow-up in March 2009, 54.2% (175 of 323) of the patients had suffered tumor recurrence, and 51.1% (165 of 323) of the patients had died. The OS rates at 1, 3, and 5 years, as well as the cumulative recurrence rates for the entire cohort, were 85.4% and 25.4%, 62.2% and 50.2%, and 50.7% and 59.7%, respectively. Notably in the high m6A expression group, the OS rates at 1, 3, and 5 years were significantly lower compared to those in the low m6A expression group (79.5% versus 89.9%, 55.7% versus 68.8%, and 42.1% versus 60.1%, respectively; Fig. 1b). Moreover, the rates of RFS at 1, 3, and 5 years were notably lower in the m6A-high group compared to the m6A-low group (65.2% versus 81.3%, 41.3% versus 55.1%, and 36.9% versus 44.5%, respectively; Fig. 1b). Both univariate and multivariate analyses revealed that m6A, alongside tumor diameter, microvascular invasion, and tumor encapsulation, acted as an independent prognostic factor for both OS (HR = 1.462, 95% CI, 1.052–2.031, *P* = 0.024) and TTR (HR = 1.422, 95% CI, 1.044–1.937, *P* = 0.025; Supplementary Table S4). In conclusion, these findings suggest that heightened m6A expression in tumors correlates with tumor differentiation, increased tumor invasiveness, and poorer long-term prognosis.

### Loss of FTO is responsible for the aberrant m6A modifications in HCC

**Fig. 2:**
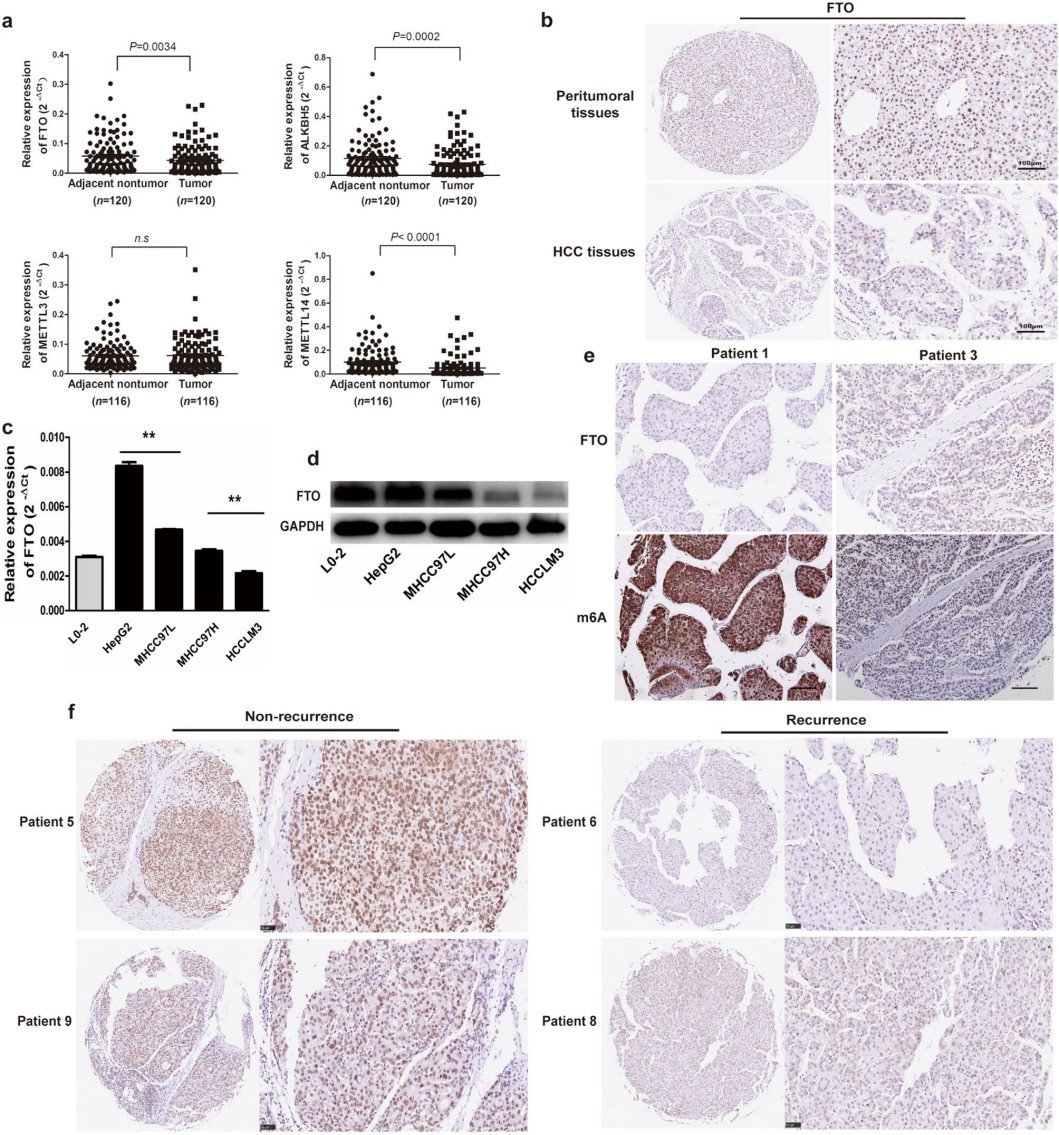
Loss of FTO is responsible for the aberrant m6A modifications in HCC. (**a**) The expression of FTO, ALKBH5, METTL3, and METTL14 were assessed in 120 pairs of HCC tissues (Cohort 1) by qRT-PCR. The expression of FTO, ALKBH5, and METTL14 in tumor samples was significantly lower compared to adjacent nontumor tissues. Statistical analysis was performed using a paired Student’s t-test. (**b**) Representative histology of FTO IHC staining in peritumor and HCC tissues. FTO predominantly localizes in the cell nucleus. (**c**) qRT-PCR revealed that the FTO expression in MHCCLM3 and MHCC97H cells, possessing high metastatic potential, was markedly lower compared to HepG2 and MHCC97L cells. Differences were assessed using an unpaired Student’s t-test. (**d**) Western blot results were consistent with the qRT-PCR findings, showing significantly lower expression levels of FTO in MHCC97H and MHCCLM3 cells compared to HepG2 and MHCC97L cells. Differences were assessed using an unpaired Student’s t-test. (**e**) There is a clear negative correlation between FTO expression and m6A levels. In the tumor tissue of Patient 1, where FTO expression is nearly absent, m6A is significantly present, while in tumor tissue from Patient 3 with high FTO expression, m6A levels are markedly diminished. (**f**) HCC patients with low expression levels of FTO have a higher tumor recurrence rate. Herein, exemplifying Patient 5 and 9 with tumor remission and Patient 6 and 8 with tumor recurrence. Data are shown as mean ± S.D. (*n* = 3). *P<0.05, **P<0.01, and ***P<0.001. Scale bars = 100 μm. HCC, hepatocellular carcinoma

The methylation process underlying m6A modifications primarily depends on the activity of methyltransferase or demethylase proteins. Disturbances in the crucial m6A methyltransferases (METTL3 and METTL14) and demethylases (FTO and ALKBH5) disrupt the regular m6A modifications. In this research, we initially evaluated the expression of methyltransferase (METTL3 and METTL14) and demethylase (FTO and ALKBH5) genes in 120 pairs of HCC tissues (cohort 1, *n* = 120) by qRT-PCR. Intriguingly, FTO, ALKBH5 and METTL14, known for their opposing roles in m6A catalysis, exhibited significant downregulation in HCC compared to the corresponding adjacent non-tumorous tissues (Fig. 2a). Conversely, the expression of METTL3 showed no significant difference between HCC and adjacent non-tumorous tissues (Fig. 2a). Immunohistochemical analyses revealed predominant nuclear localization of FTO and reduced expression in tumor compared to the paired peritumoral tissues (Fig. 2b).

Subsequently, we accessed the expression of FTO across various human HCC cell lines. Our results revealed a notable reduction in FTO expression within highly metastatic cell lines (MHCC97H and HCCLM3) compared to less metastatic HCC cell lines (HepG2 and MHCC97L) at both the mRNA (*P* < 0.01, Fig. 2c) and protein levels (Fig. 2d). Immunohistochemical analyses depicted an inverse correlation between FTO expression and m6A levels, highlighting the impact of FTO deficiency on increased m6A levels in HCC tissues (Fig. 2e). Additionally, diminished FTO levels were observed in patients with HCC recurrence compared to those without recurrence (Fig. 2f). These cumulative findings underscore the pivotal role of FTO in the aberrant m6A modifications observed in HCC. FTO levels were frequently observed to be downregulated in HCC, particularly in patients experiencing HCC recurrence, suggesting a potential implication of FTO in HCC metastasis and recurrence via modulation of m6A modification.

### Forced expression of FTO attenuates HCC growth and metastasis

**Fig. 3:**
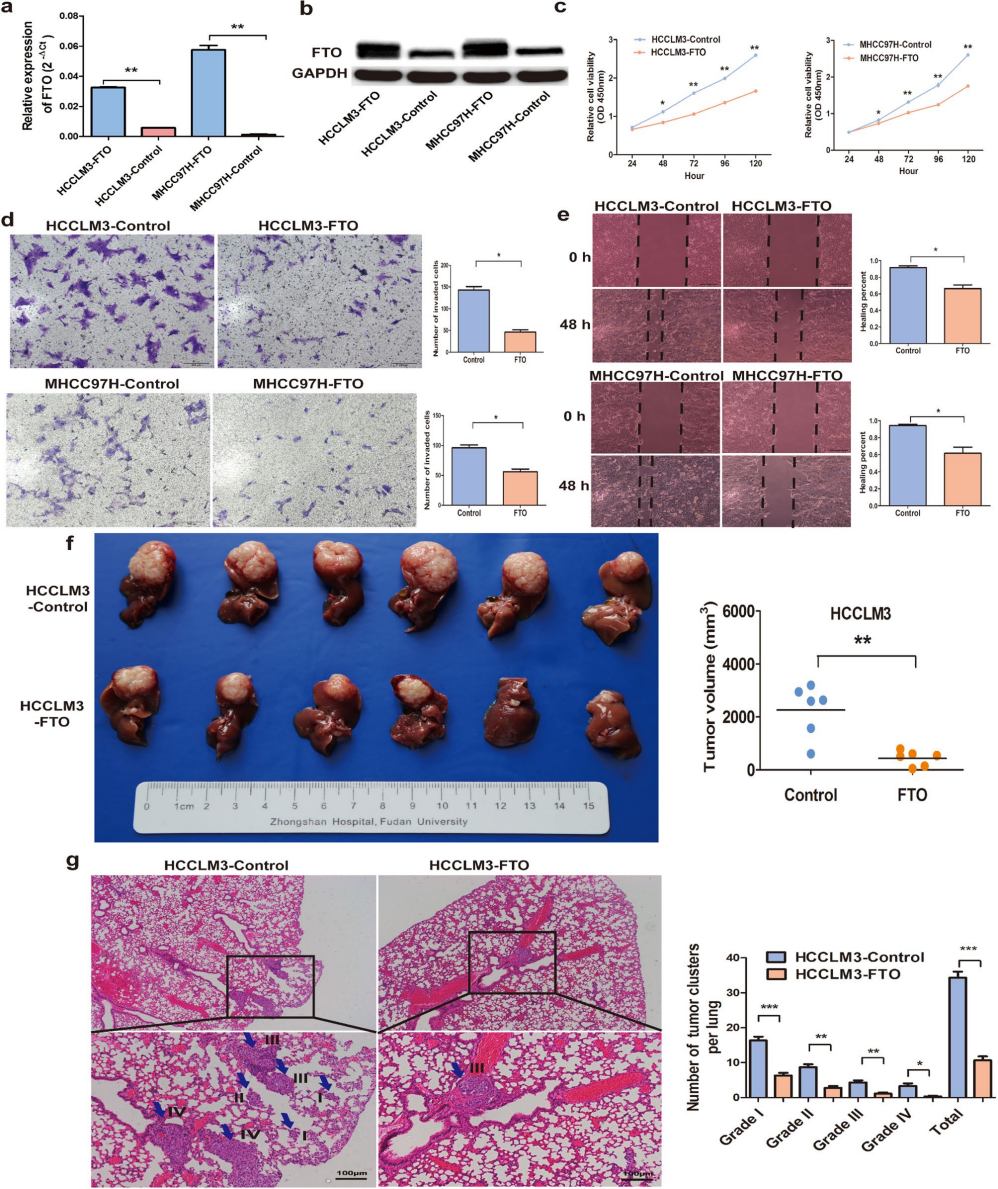
Forced expression of FTO attenuates HCC growth and metastasis. Endogenous FTO mRNA (**a**) and protein (**b**) levels in MHCC97H and MHCCLM3 cells were increased following the introduction of the FTO lentiviral vectors. The proliferative capacity (**c**), invasive capacity (**d**) and migratory ability (**e**) of MHCC97H and MHCCLM3 cells exhibiting FTO overexpression experienced a notable decrease compared with the negative controls. (**f**) The growth rate of FTO-overexpressing MHCCLM3 cells in mice liver was significantly lower than that of the control group. (**g**) FTO-overexpressing MHCCLM3 cells exhibited a significantly reduced ability to metastasize to the lungs in mice, with the control group showing a higher number of lung metastases compared to the overexpressing group. All differences were assessed using unpaired Student’s t-test. Data are shown as mean ± S.D. (*n* = 3). *P<0.05, **P<0.01, and ***P<0.001. Scale bars = 100 μm. HCC, hepatocellular carcinoma

To delve deeper into the impact of FTO on tumor proliferation, invasion, and metastasis, we proceeded to elevate endogenous FTO levels in MHCC97H and MHCCLM3 cells by employing FTO lentiviral vectors, thereby establishing HCC cell lines with stably overexpressed FTO. The augmentation of FTO expression was validated at both the mRNA and protein levels (Fig. 3a and b). The overexpression of FTO led to a modest yet significant suppression of the proliferation rate of HCC cells under low-serum (1%) culture conditions (Fig. 3c). Notably, cell migration assays revealed that FTO overexpression compromised the migratory and invasive capabilities of HCC cells (Fig. 3d). Specifically, in migration transwell assays, FTO overexpression significantly impeded the migratory potential of MHCC97H and MHCCLM3 cells (Fig. 3d). Furthermore, wound-healing migration assays demonstrated a noticeable deceleration in the closure rate of wounds in MHCC97H-FTO and HCCLM3-FTO cells compared to the control cell lines (Fig. 3e).

Given the significant reduction in cell invasion observed in vitro following FTO overexpression in HCCLM3 cells, we proceeded to establish orthotopic HCC mouse models to explore the impact of FTO in vivo. Comparison with the nontarget control group revealed a notable suppression of HCC growth in nude mice upon FTO overexpression. The tumor size underwent a substantial reduction upon FTO overexpression (2259.0 ± 980.4 mm^3^ versus 438.9 ± 283.1 mm^3^, *P* = 0.006; Fig. 3f). Furthermore, we observed a significant attenuation of HCC lung metastasis in vivo following FTO overexpression. The number of metastatic nodules of each grade is depicted in Fig. 3g. The pulmonary metastasis rate in HCCLM3-FTO mice was 33.3% (2/6), whereas in HCCLM3-control mice, it was 100% (6/6). Overall, our findings strongly suggest that FTO plays a pivotal role in suppressing HCC growth and metastasis.

### Loss of FTO enhances HCC invasion and metastasis

**Fig. 4:**
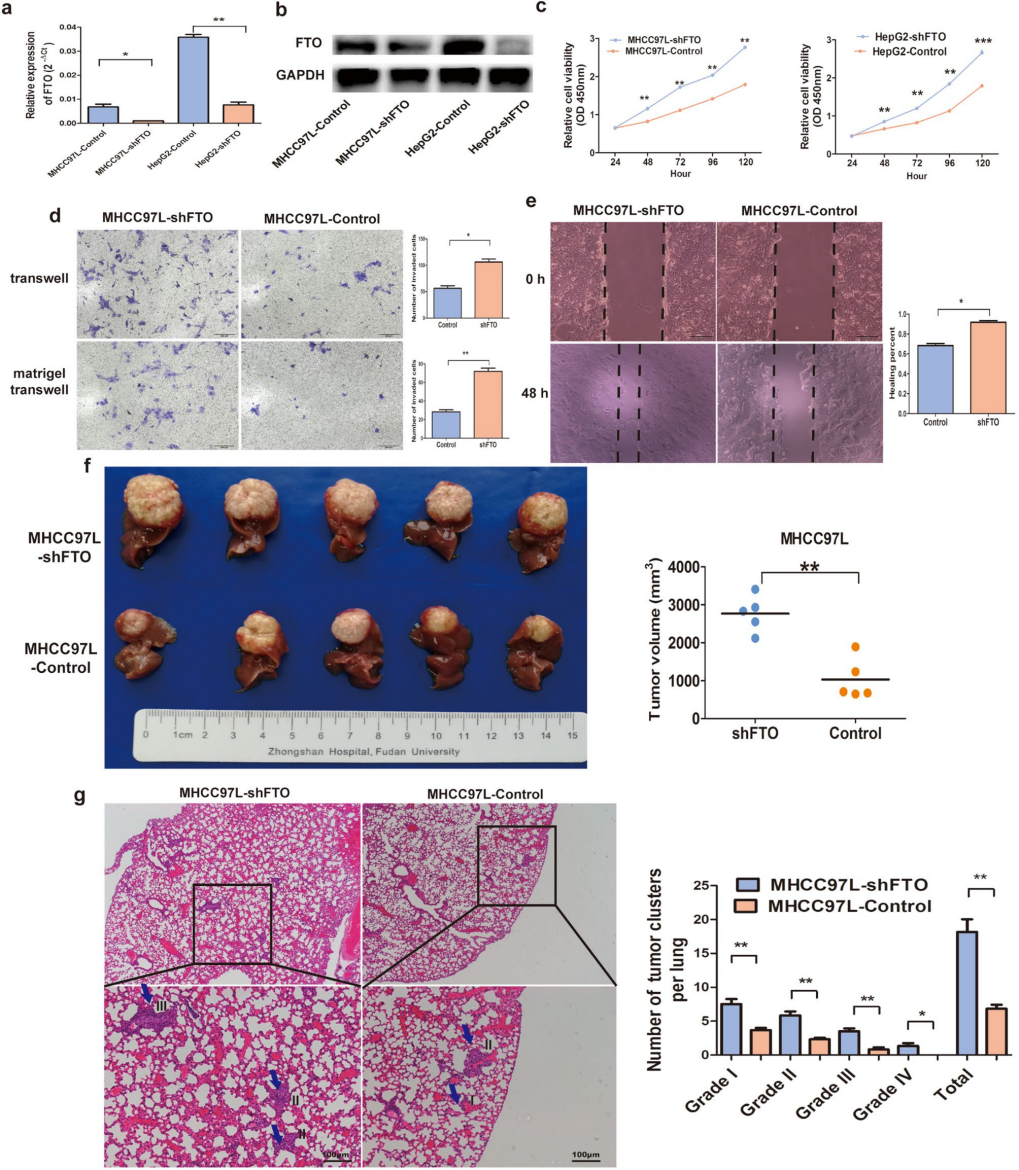
Loss of FTO enhances HCC invasion and metastasis. (**a**) Endogenous FTO mRNA levels in MHCC97L and HepG2 cells were reduced through the FTO shRNA. (**b**) FTO protein expression was also decreased following the introduction of FTO shRNA. (**c**) The proliferation rate of MHCC97L and HepG2 cells with FTO knockdown was significantly accelerated compared to the negative control. (**d**) The invasive and migratory capacity of MHCC97L cells with FTO knockdown was notably enhanced compared to the negative control. (**e**) The migratory ability of MHCC97L and HepG2 cells with FTO knockdown exhibited a marked increase compared to the negative control. (**f**) The growth rate of MHCC97L-shFTO cells in mice liver tumors was significantly higher than that of the negative control. (**g**) MHCC97L-shFTO cells showed a significantly enhanced ability to metastasize to the lungs in mice, with the control group exhibiting a lower number of lung metastases compared to the FTO-knockdown cells. All differences were assessed using an unpaired Student’s t-test. Data are shown as mean ± S.D. (*n* = 3). *P<0.05, **P<0.01, and ***P<0.001. Scale bars = 100 μm. HCC, hepatocellular carcinoma

Subsequently, we employed shRNA to downregulate FTO in two HCC cell lines, MHCC97L and HepG2. The effective knockdown of FTO was confirmed at both the mRNA and protein levels (Fig. 4a and b). This FTO knockdown markedly intensified the proliferation of HCC cells (Fig. 4c). Furthermore, cell migration and invasion assays revealed that the downregulation of FTO promoted the migratory and invasive capabilities of HCC cells. In migration transwell assays, the knockdown of FTO enhanced the migration ability of MHCC97L cells (56.3 ± 8.1 versus 106.3 ± 9.6, *P* = 0.011; Fig. 4d). Consistent with the non-matrigel migration assays, FTO knockdown in MHCC97L cells resulted in an augmented invasive ability compared to the control cell lines (28.3 ± 4.0 versus 72.0 ± 6.0, *P* = 0.004; Fig. 4d). Wound-healing migration assays demonstrated an accelerated rate of wound closure of MHCC97L-shFTO cells compared to the control cell lines (Fig. 4e).

An in vivo orthotopic implantation experiment was undertaken to further affirm the impact of FTO knockout on HCC tumorigenicity. It was observed that the knockout of FTO significantly enhanced tumor formation within the orthotopic liver microenvironment (Fig. 4f) and strengthened lung metastasis in nude mice (Fig. 4g). Comparative analysis revealed a significant increase in tumor size in the MHCC97L-shFTO group compared to the control group (2768.0 ± 476.2 mm^3^ versus 1032.0 ± 538.7 mm^3^, *P* = 0.008; Fig. 4f). Additionally, a higher occurrence of micro-metastases was observed in the FTO knockdown group, as illustrated in Fig. 4g. The pulmonary metastasis rate in MHCC97L-shFTO mice was 100% (5/5), whereas in MHCC97L-control mice, it was 40.0% (2/5), suggesting that FTO depletion could promote lung metastasis (Fig. 4g). Overall, these findings elucidate that FTO silencing amplifies tumor invasion and metastasis in HCC.

### Loss of FTO indicated poor prognosis for HCC patients

**Fig. 5:**
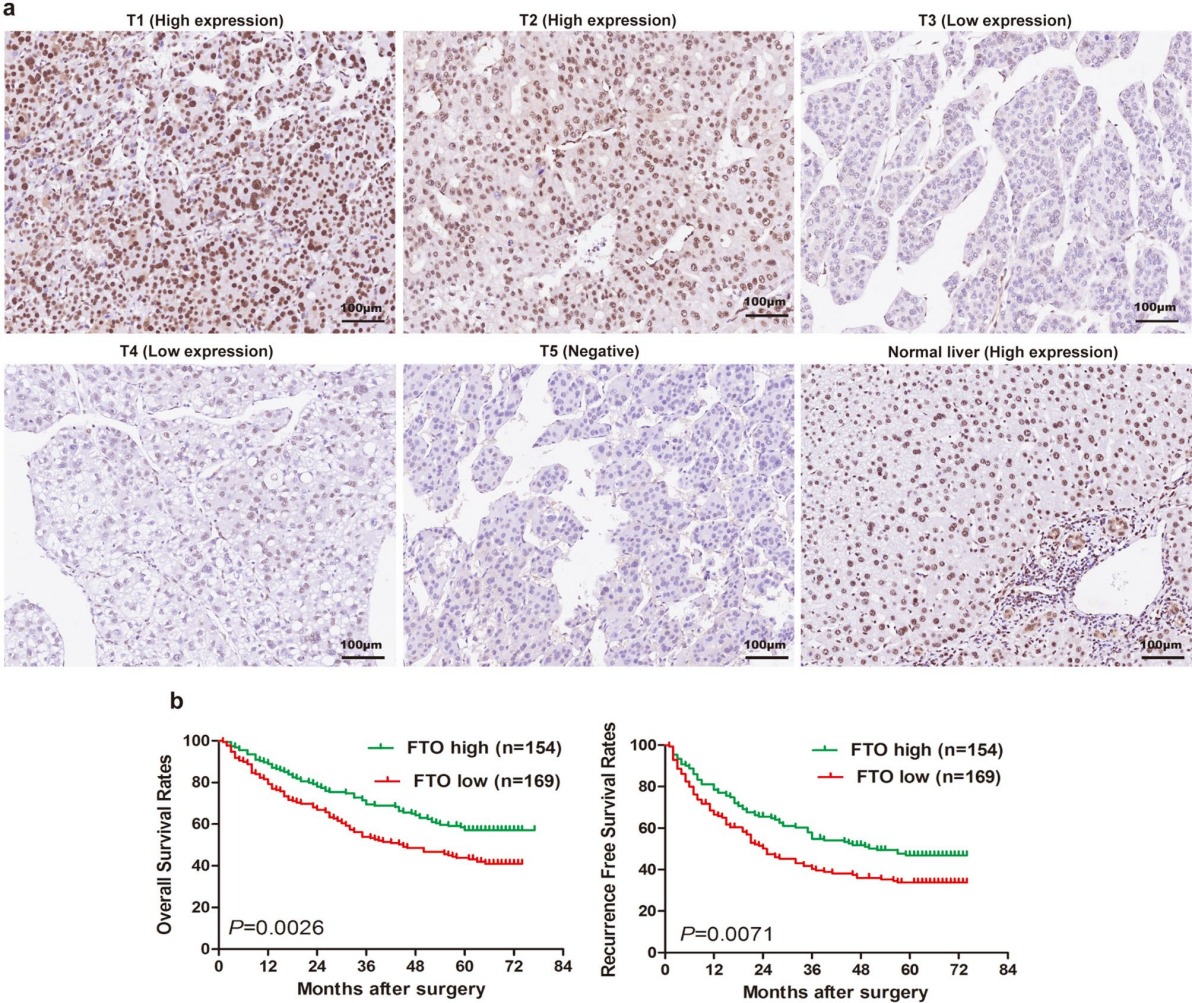
Loss of FTO indicated poor prognosis for HCC patients. (**a**) Based on the intensity of FTO expression levels in the nucleus as determined by immunohistochemistry, it was categorized into T1-T5, where T1 and T2 were defined as the high expression group, and T3-T5 were defined as the low expression group. (**b**) Patients with high FTO expression in tumor cells exhibited improved overall survival and recurrence-free survival (cohort 2, *n* = 323). Group differences were assessed using the Kaplan-Meier method. HCC, hepatocelluar carcinoma

FTO protein expression in primary tumors from 323 HCC patients was assessed using immunohistochemistry on a tissue microarray (cohort 2, *n* = 323). It was observed that FTO-positive staining predominantly localized in cell nuclei. While normal liver cells consistently exhibited high expression of FTO, HCC cells showed a notable downregulation of FTO expression (Fig. 5a). The correlations between FTO expression and clinicopathologic parameters were summarized in Supplementary Table S5. Our findings revealed significant associations between FTO expression and sex (*P* = 0.026), GGT (*P* = 0.009), tumor size (*P* = 0.034), microvascular invasion (*P* = 0.001), and tumor differentiation (*P* < 0.001, Supplementary Table S5). However, clinical characteristics such as age, hepatitis history, AFP, liver cirrhosis, multiple tumors, tumor encapsulation, and TNM stage were not directly linked to FTO expression (Supplementary Table S5).

Elevated FTO levels exhibited significant correlations with prolonged OS (*P* = 0.003) and RFS (*P* = 0.007, Fig. 5b). Kaplan-Meier survival analysis demonstrated that patients with high FTO levels had notably higher 1-, 3-, and 5-year survival rates compared to those with low FTO levels, at 89.0% versus 79.3%, 69.5% versus 53.8%, and 57.1% versus 43.8%, respectively (*P* = 0.002, Fig. 5b). Similarly, patients with high FTO levels displayed significantly higher 1-, 3-, and 5-year RFS rates (78.4%, 54.9%, and 46.9%, respectively) compared to those with low FTO levels (66.5%, 40.3%, and 33.8%, respectively; *P* = 0.007, Fig. 5b). Furthermore, the multivariate analyses indicated that FTO served as an independent prognostic factor for OS (HR = 0.695, 95% CI, 0.504–0.957, *P* = 0.026; Table 1), along with GGT, tumor size, microvascular invasion, tumor encapsulation, and tumor differentiation (Table 1). Additionally, our multivariate analysis revealed that sex, tumor size, microvascular invasion, and tumor encapsulation were independent prognostic factors for TTR (Table 1).

**Table 1:** Univariate and multivariate analyses of prognosis in hepatocellular carcinoma (cohort 2, *n* = 323)

Variable	Time to recurrence		Overall survival	
	HR (95% CI)	*P*	HR (95% CI)	*P*
**Univariate analysis**				
Age, year (≤ 50 versus > 50)	0.977(0.726–1.315)	0.879	1.207(0.889–1.640)	0.228
Sex (female versus male)	1.863(1.143–3.036)	**0.013**	1.757(1.049–2.942)	**0.032**
HBsAg (negative versus positive)	0.978(0.664–1.442)	0.912	1.002(0.670–1.498)	0.993
AFP, ng/ml (≤ 20 versus > 20)	1.155(0.835–1.597)	0.385	1.548(1.083–2.211)	**0.016**
GGT, U/L (≤ 54 versus > 54)	1.329(0.978–1.805)	0.069	1.737(1.253–2.409)	**0.001**
Liver cirrhosis (no versus yes)	1.160(0.712–1.889)	0.552	1.362(0.801–2.315)	0.254
Tumor size, cm (≤ 5 versus > 5)	1.817(1.346–2.452)	**0.000**	2.482(1.806–3.412)	**0.000**
Tumor number (single versus multiple)	1.362(0.907–2.044)	0.136	1.517(1.025–2.243)	**0.037**
Microvascular invasion (no versus yes)	1.915(1.420–2.583)	**0.000**	2.479(1.815–3.388)	**0.000**
Tumor encapsulation (complete versus none)	1.679(1.246–2.263)	**0.001**	1.715(1.260–2.336)	**0.001**
Tumor differentiation (I + II versus III + IV)	1.238(0.870–1.753)	**0.236**	1.581(1.119–2.233)	**0.009**
TNM stage (I versus II + III)	1.226(0.912–1.650)	0.178	1.563(1.149–2.125)	**0.004**
FTO (low versus high)	0.667(0.494-0.900)	**0.008**	0.624(0.457–0.852)	**0.003**
**Multivariate analysis**				
Sex (female versus male)	1.740(1.066–2.842)	**0.027**	1.421(0.837–2.414)	0.193
AFP, ng/ml (≤ 20 versus > 20)	NA	NA	1.218(0.838–1.770)	0.301
GGT, U/L (≤ 54 versus > 54)	NA	NA	1.466(1.041–2.065)	**0.029**
Tumor size, cm (≤ 5 versus > 5)	1.629(1.198–2.216)	**0.002**	1.964(1.400-2.754)	**0.000**
Tumor number (single versus multiple)	NA	NA	1.309 (0.870–1.970)	0.196
Microvascular invasion (no versus yes)	1.582(1.157–2.162)	**0.004**	2.062(1.482–2.869)	**0.000**
Tumor encapsulation (complete versus none)	1.590(1.171–2.158)	**0.003**	1.656(1.202–2.281)	**0.002**
Tumor differentiation (I -II versus III + IV)	NA	NA	1.865(1.311–2.655)	**0.001**
TNM stage (I versus II + III)	NA	NA	NA	NA
FTO (low versus high)	0.774(0.567–1.056)	0.106	0.695(0.504–0.957)	**0.026**

Analyses were conducted using univariate analysis or multivariate Cox proportional hazards regression

AFP, alpha-fetoprotein; GGT, gamma glutamyl transferase; HBsAg, hepatitis B surface antigen; FTO, fat mass and obesity-associated protein; TNM, tumor-node-metastasis; HR, hazard ratio; CI, confidential interval; NA, not adopted

### FTO inhibits HCC by downregulating VEGFA expression

**Fig. 6:**
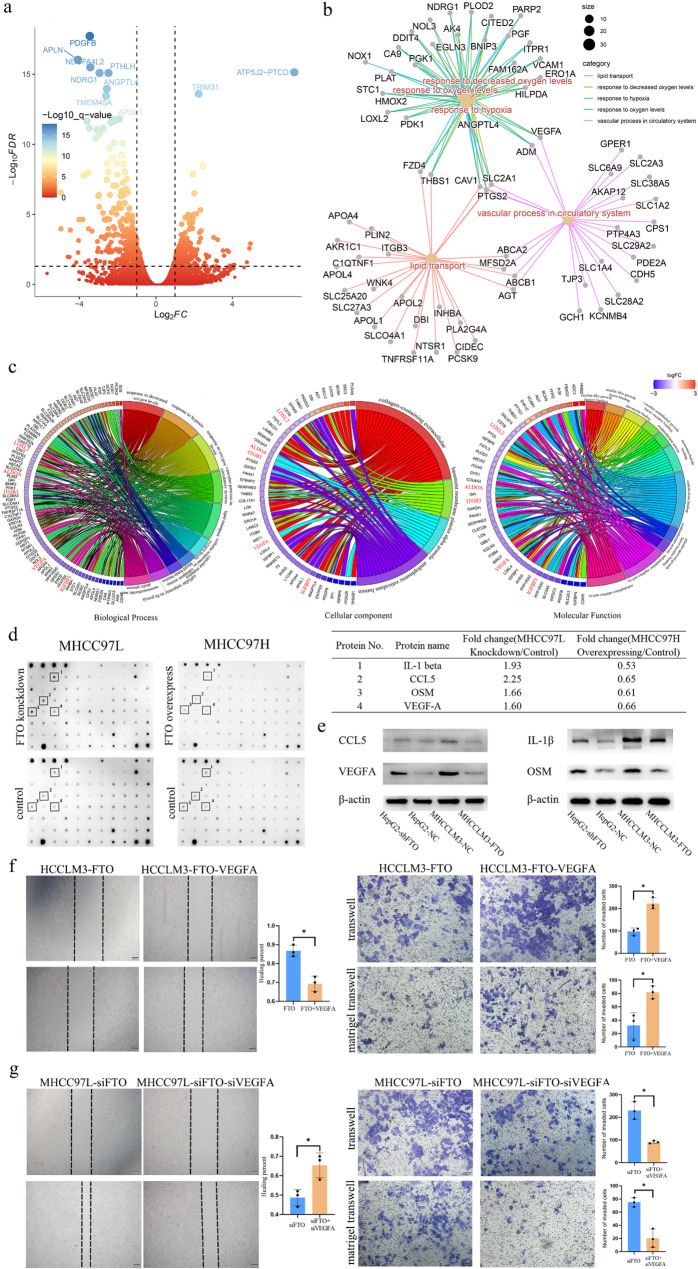
FTO inhibits HCC by downregulating VEGFA expression. (**a**) Transcriptional sequencing of FTO-overexpressing MHCCLM3 cells compared to the control group revealed significant downregulation of 309 genes and upregulation of 227 genes. Differentially expressed genes were computed using the negative binomial generalized linear model in edgeR. (**b**) GO analysis of the differentially expressed genes unveiled ten major functional pathways and 78 key genes. (**c**) Further analysis indicated that the cellular biological processes primarily affected by FTO overexpression were lipid metabolism, angiogenesis, and hypoxia. (**d**) Results from protein chip detection showed an inverse trend between VEGFA expression and FTO alteration. Knockdown of FTO in MHCC97L cells led to upregulation of VEGFA, while overexpression of FTO in MHCC97H cells resulted in significant downregulation of VEGFA. Differences were assessed using an unpaired Student’s t-test. (**e**) Western blot analysis indicated a significant upregulation of VEGFA expression in FTO-knockdown HepG2 cells, whereas FTO-overexpressing MHCCLM3 cells exhibited a pronounced downregulation of VEGFA expression. (**f**) We reversed of the FTO-induced downregulation of VEGFA expression through VEGFA plasmid. The results of the scratch assay and transwell assay demonstrated that the invasive and migratory abilities of HCCLM3 were significantly enhanced after the restoration of VEGFA expression. (**g**) VEGFA expression was reduced in FTO-upregulated 97 L cells through VEGFA siRNA, and the results of the scratch assay and transwell assay revealed a corresponding decrease in the invasive and migratory abilities of 97 L cells. Differences were assessed using an unpaired Student’s t-test. *P<0.05

To further elucidate the mechanism by which FTO regulates the biological behavior of HCC, RNA-Seq sequencing was conducted on MHCCLM3-FTO and MHCCLM3-NC cells. Among over 25,000 genes, FTO overexpression significantly downregulated 300 genes and upregulated 210 genes (Fig. 6a, fold change ≥ 2, *P* < 0.01). To identify key genes regulated by FTO that impact HCC cell behavior, Gene Ontology analysis of the differentially expressed genes was further conducted. The functional enrichment primarily focused on hypoxia, angiogenesis, and lipid metabolism, with 78 genes playing a major role in biological processes (Fig. 6b). While previous studies have confirmed the crucial role of FTO in lipid metabolism, there have been no reports regarding its involvement in hypoxia and angiogenesis. Notably, hypoxia and angiogenesis are both significant components in tumor development. The expression of differentially expressed genes combined with the functional pathway association network revealed that VEGFA, ADM, CAV1, PTGS2 and SLC2A1 (Fig. 6c) were key genes in biological process, cellular component and molecular function. VEGFA induces endothelial cell proliferation and migration and is indispensable for tumor vascularization. We assessed these genes in the HCCDB database, VEGFA is not only clearly associated with hepatocellular carcinoma prognosis, but is primarily secreted by malignant cells (Supplementary Fig. 1). Therefore, it was speculated that FTO could be a key regulator in HCC development.

Additionally, through protein chip analysis, changes in cytokine secretion levels following FTO expression in MHCC97L and MHCC97H were detected. The results revealed that the expression of VEGFA, CCL5, IL1B, and OSM is reduced with increased FTO levels, and vice versa (Fig. 6d and Supplementary Table S6). These cytokine expression levels were further validated in HepG2 and MHCCLM3 cells through western blot, demonstrating consistent results with the protein chip analysis, as the expression levels of VEGFA and FTO exhibit opposite trends (Fig. 6e). In conclusion, it was discovered that the negative regulation of VEGFA expression is a key mechanism through which FTO inhibits the invasion and metastasis of HCC. We reversed the effects of FTO on VEGFA expression in hepatocellular carcinoma cells through VEGFA siRNA or plasmids. the results of the scratch assay and transwell assay showed that after antagonizing the downregulation of VEGFA by FTO in HCCLM3 cells, tumor invasiveness and migratory capacity were significantly upregulated (Fig. 6f). In contrast, the opposite was observed in MHCC97L cells; in MHCC 97 L cells with suppressed FTO expression, inhibiting VEGFA expression led to a corresponding decrease in cell invasiveness and migratory ability (Fig. 6g).

## Discussion

The intricate relationship between FTO, m6A modification, and HCC underscores a complex interplay. Dysregulated FTO expression leads to altered m6A methylation patterns, exacerbating the invasive characteristics of HCC cells. Understanding these molecular interactions offers promising avenues for targeted therapeutic interventions in HCC treatment. In this study, we discovered that FTO negatively regulates VEGFA expression through m6A methylation in HCC, thereby inhibiting angiogenesis and consequently suppressing the invasion and metastasis of HCC.

FTO is the first obesity susceptibility gene identified through GWAS and represents the locus with the greatest impact on body mass index and obesity risk [4, 5]. Previous research has demonstrated that FTO SNP influences individuals’ susceptibility to obesity through subtle effects on diet and energy intake [23, 24], and subsequently leads to a range of obesity-related complications, such as type 2 diabetes [25]. HCC is one of the tumors most closely linked to obesity and diabetes [26], and diabetes is one of the significant independent risk factors for the prognosis of HCC [27]. However, the relationship between FTO and HCC does not stop there; research has demonstrated that obesity-induced hepatic steatosis, fibrosis, and HCC can be three separate processes [3]. Therefore, we hypothesize that the expression of FTO directly affects the biological behavior of HCC cells, rather than through indirect effects of obesity or fatty liver. FTO is the first identified m6A demethylase, and we have found that the levels of m6A in HCC tissues are closely associated with prognosis. Furthermore, significant correlations were observed between FTO and m6A alterations. Subsequent research has also confirmed that FTO expression directly alters the invasive and metastatic abilities of HCC cells. It is thus apparent that FTO serves as a direct driving force of HCC, rather than an indirect influence akin to its effects on obesity and diabetes.

Previous studies have reported on certain genes influencing the biological behavior of HCC through FTO, but there has been minimal exploration into the downstream effects of FTO. Through various methods such as transcriptome sequencing and protein microarrays, we confirmed the negative regulatory effect of FTO on VEGFA expression. Interestingly, while the role of m6A modification in the regulation of VEGFA expression and function has been extensively studied, the regulation of VEGFA by FTO has not been reported. The m6A “writers” METTL3 and m6A “readers” IGF2BP2/3 were found to participate in the methylation of VEGFA to prevent mRNA degradation, and to promote angiogenesis in colorectal cancer by mimicking the formation of the PI3K/AKT/mTOR and ERK1/2 signaling pathways [28]. This pathway also regulates VEGFA expression in bladder cancer [29]. Further research has revealed that METTL3 alters the expression of VEGFA splice variants, vegfa-164 and vegfa-188, in Bone Marrow Mesenchymal Stem Cells [30]. In lung cancer, the YTHDC2/eIF4GI complex triggers cap-independent translation initiation, and targeted specific demethylation of VEGFA m6A significantly reduced VEGFA expression [31]. Additionally, in colorectal cancer, WTAP activates the MAPK signaling pathway by mediating m6A methylation in VEGFA mRNA via YTHDC1, promoting tumor progression [32]. WTAP also recruits macrophages and increases VEGFA secretion through N6-methyladenosine modification in corneal neovascularization [33]. In addition to the aforementioned m6A-related genes, it has been discovered for the first time that FTO can also regulate alterations in VEGFA. However, public data (GSE178095, GSE171472 and GSE128582) suggests that changes in FTO expression have not significantly altered the m6A modification of VEGFA, indicating that FTO may regulate VEGFA through other mechanisms.

The altered expression of FTO in HCC in this study primarily affected vascularization, hypoxia, and lipid metabolism. There has been a plethora of research on FTO and lipid metabolism in the past [34]. Vascularization and hypoxia are both key driving factors in the development of HCC, and the results of bioinformatics analysis indicate that the genes VEGFA and ADM play crucial roles in these physiological processes. Subsequently, we further validated the correlation between VEGFA expression and FTO expression through protein microarrays and western blot. FTO was found to have a clear negative regulatory effect on the secretion of the VEGFA cytokine. These findings suggest that FTO influences the invasion and metastasis of HCC through its regulation of vascularization.

As early as 1939, the scientists speculated that tumors could release angiogenic factors to promote angiogenesis [35]. The first VEGFA inhibitor, bevacizumab, was introduced in 2004 and used in the treatment of colorectal cancer [36]. In HCC, angiogenesis inhibitors have become almost the sole treatment pathway for advanced-stage HCC. Among these, the combination of bevacizumab with PD-1 antibodies has emerged as the first-line standard therapy for advanced-stage HCC [37]. The success of angiogenesis inhibitors has confirmed VEGFA as a pivotal clinical target, crucial for patients with late-stage cancers who have limited treatment options. However, there is significant heterogeneity in patient responses to treatment, with objective response rates of various angiogenesis inhibitors in HCC being less than 50% [38]. Therefore, the search for specific predictive biomarkers remains a crucial issue, with no validated biomarkers associated with VEGFA inhibitors in HCC thus far. In clinical practice, the utilization of VEGFA inhibitors remains largely empirical, lacking robust reference standards. In this study, FTO has demonstrated a distinct regulatory role on VEGFA. It may potentially serve as a predictive factor for the efficacy of VEGFA inhibitors such as bevacizumab and ramucirumab. This holds crucial significance for alleviating the suffering imposed by targeted therapy on advanced HCC patients and enhancing their prognosis, yet further subsequent investigations are warranted.

## Conclusion

In conclusion, the expression level of FTO directly affects the invasive ability of HCC cells and can serve as a prognostic marker for long-term outcomes in HCC patients. FTO can inhibit vascularization and consequently suppress the progression of HCC by negatively regulating the expression of VEGFA. The expression of FTO could potentially serve as a novel therapeutic target or predictive indicator for drug resistance in HCC.

## Electronic supplementary material

Below is the link to the electronic supplementary material.

Supplementary Material 1

Supplementary Material 2: Fig. 1. VEGFA levels in hepatocellular carcinoma are significantly correlated with the prognosis (A) and are primarily secreted by malignant cells rather than immune or stromal cells (B).

Supplementary Material 3

## Data Availability

The datasets used and/or analysed during the current study are available from the corresponding author on reasonable request.

## Abbreviations

ALDOA: Aldolase A
ALKBH5: AlkB homolog 5
CCL5: C-C motif chemokine ligand 5
FTO: Fat mass and obesity-associated protein
GWAS: Genome-wide Association Study
HCC: Hepatocellular Carcinoma
IGFBP3: Insulin-like Growth Factor Binding Protein 3
IL1B: Interleukin 1 beta
ITGB3: Integrin Subunit Beta 3
LOXL2: Lysyl Oxidase-like 2
METTL14: Methyltransferase like 14
METTL3: Methyltransferase like 3
m6A: N6-methyladenosine
NAFLD: Nonalcoholic fatty liver disease
NASH: Nonalcoholic steatohepatitis
OS: Overall survival
OSM: Oncostatin M
RFS: Recurrence-free survival
TTR: Time to recurrence
VEGFA: Vascular Endothelial Growth Factor A
