# Supplementary material for: FTO downregulation-mediated m6A modification resulting in enhanced hepatocellular carcinoma invasion
Source: Cell Biosci. 2025 May 2;15:58. doi: 10.1186/s13578-025-01395-w (PMC12049069; doi:10.1186/s13578-025-01395-w)
Supplement: Supplementary file 1 — Supplementary Material 1 [file 13578_2025_1395_MOESM1_ESM.docx]

Supplementary Table S1. The primer sequences of human RNAs for real-time PCR

| \| Primer name and sequences \| \| \| --- \| --- \| \| FTO \| Forward: 5’-GTGACAAATGCTGTGCTTCA-3’ \| \| Reverse: 5’-CAGGTAATGTTCGGGCAAT-3’ \| \| ALKBH5 \| Forward: 5’-CCAGCGTGGATTTCTCAAGT-3’ \| \| Reverse: 5’-ACATATCAGGGCGAAGTGCT-3’ \| \| mettl3 \| Forward: 5’-ATGGGAAGGAACACTGCTTG-3’ \| \| Reverse: 5’-ATGACTGGTGGAACGAACCT-3’ \| \| mettle14 \| Forward: 5’-GCATCCTATTTCAGTGCTCCT-3’ \| \| Reverse: 5’-CACCTCTTCCTCCACCTCTG-3’ \| \| GAPDH \| Forward: 5’-AAGGTGAAGGTCGGAGTCAAC -3’ \| \| Reverse: 5’-GGGGTCATTGATGGCAACAATA-3’ \| |
| --- | --- | --- | --- | --- | --- | --- | --- | --- | --- | --- | --- | --- | --- | --- | --- | --- | --- |

Supplementary Table S2. Primary antibodies used in western blot, immunohistochemistry tests

| \| Antibody \|  \| Concentration for WB \| Concentration for IHC \| Company \| \| --- \| --- \| --- \| --- \| --- \| \| FTO \|  \| 1:1000 \| 1:500 \| Abcam \| \| M6a \|  \| / \| 1:400 \| Abcam \| \| CCL5 \|  \| 1:1000 \| / \| Cell Signaling Technology \| \| VEGFA \|  \| 1:1000 \| / \| Cell Signaling Technology \| \| IL-1β \|  \| 1:1000 \| / \| Cell Signaling Technology \| \| OSM \|  \| 1:1000 \| / \| Cell Signaling Technology \| \| β-actin \|  \| 1:1000 \| / \| Cell Signaling Technology \| \| GAPDH \|  \| 1:4000 \| / \| Abcam \| |
| --- | --- | --- | --- | --- | --- | --- | --- | --- | --- | --- | --- | --- | --- | --- | --- | --- | --- | --- | --- | --- | --- | --- | --- | --- | --- | --- | --- | --- | --- | --- | --- | --- | --- | --- | --- | --- | --- | --- | --- | --- | --- | --- | --- | --- | --- |

Supplementary Table S3. Correlation between m6A and Clinicopathologic Characteristics in Hepatocellular Carcinoma (cohort 2, *n*=323)

| Clinicopathological Indexes | | m6A | | *P* |
| --- | --- | --- | --- | --- |
|  |  | Low | High |  |
| Age(year) | ≤ 50 | 61 | 105 | **0.017** |
|  | >50 | 77 | 80 |  |
| Sex | Female | 18 | 28 | 0.632 |
|  | Male | 120 | 157 |  |
| HBsAg | Negative | 24 | 21 | 0.144 |
|  | Positive | 114 | 164 |  |
| AFP (ng/ml) | ≤20 | 48 | 48 | 0.109 |
|  | >20 | 90 | 137 |  |
| GGT (U/L) | ≤54 | 61 | 70 | 0.255 |
|  | >54 | 77 | 115 |  |
| Liver cirrhosis | No | 21 | 16 | 0.078 |
|  | yes | 117 | 169 |  |
| Tumor size(cm) | ≤5 | 66 | 98 | 0.370 |
|  | >5 | 72 | 87 |  |
| Tumor number | Single | 115 | 161 | 0.425 |
|  | Multiple | 23 | 24 |  |
| Microvascular invasion | absence | 86 | 89 | **0.013** |
|  | present | 52 | 96 |  |
| Tumor encapsulation | complete | 79 | 85 | 0.056 |
|  | none | 59 | 100 |  |
| Tumor differentiation | I+II | 117 | 139 | **0.038** |
|  | III+IV | 21 | 46 |  |
| TNM stage | I | 78 | 86 | 0.091 |
|  | II+III | 60 | 99 |  |

Fisher’s exact tests or chi-square tests for all analyses. P values less than 0.05 were considered statistically significant. Boldface type indicates significant values. AFP, alpha-fetoprotein; GGT, gamma glutamyl transferase; HBsAg, hepatitis B surface antigen; m-6-A, N6-methyladenosine; TNM, tumor-node-metastasis.

| Variable | OS | |  | TTR | |
| --- | --- | --- | --- | --- | --- |
|  | HR (95% CI) | *P* |  | HR (95% CI) | *P* |
| Univariate analysis |  |  |  |  |  |
| Age, year (≤50 versus >50) | 0.828(0.610-1.125) | 0.228 |  | 1.023(0.760-1.377) | 0.879 |
| Sex (female versus male) | 1.757(1.049-2.942) | **0.032** |  | 1.863(1.143-3.036) | **0.013** |
| HBsAg (negative versus positive) | 1.002(0.670-1.498) | 0.993 |  | 0.978(0.664-1.442) | 0.912 |
| AFP, ng/ml (≤20 versus>20) | 1.548(1.083-2.211) | **0.016** |  | 1.155(0.835-1.597) | 0.385 |
| GGT, U/L (≤54 versus >54) | 1.737(1.253-2.409) | **0.001** |  | 1.329(0.978-1.805) | 0.069 |
| Liver cirrhosis (no versus yes) | 1.362(0.801-2.315) | 0.254 |  | 1.160(0.712-1.889) | 0.552 |
| Tumor size, cm (≤5 versus>5) | 2.482(1.806-3.412) | **0.000** |  | 1.817(1.346-2.452) | **0.000** |
| Tumor number (single versus multiple) | 1.517(1.025-2.243) | **0.037** |  | 1.362(0.907-2.044) | 0.136 |
| Microvascular invasion (no versus yes) | 2.479(1.815-3.388) | **0.000** |  | 1.915(1.420-2.583) | **0.000** |
| Tumor encapsulation (complete versus none) | 1.715(1.260-2.336) | **0.001** |  | 1.679(1.246-2.263) | **0.001** |
| Tumor differentiation (I + II versus III + IV) | 1.581(1.119-2.233) | **0.009** |  | 1.238(0.870-1.753) | 0.236 |
| TNM stage (I versus II+ III) | 1.563(1.149-2.125) | **0.004** |  | 1.226(0.912-1.650) | 0.178 |
| m-6-A (low versus high) | 1.666(1.208-2.296) | **0.002** |  | 1.387(1.025-1.877) | **0.034** |
| Multivariate analysis |  |  |  |  |  |
| Sex (female versus male) | 1.519(0.902-2.558) | 0.116 |  | 1.857(1.138-3.031) | **0.013** |
| AFP, ng/ml (≤20 versus>20) | 1.301(0.903-1.875) | 0.157 |  | NA | NA |
| GGT, U/L (≤54 versus >54) | 0.721(0.510-1.019) | 0.064 |  | NA | NA |
| Tumor size, cm (≤5 versus>5) | 1.970(1.383-2.804) | **0.000** |  | 1.718(1.249-2.362) | **0.001** |
| Tumor number (single versus multiple) | 1.245(0.831-1.865) | 0.288 |  | NA | NA |
| Microvascular invasion (no versus yes) | 1.974(1.425-2.734) | **0.000** |  | 1.461(1.062-2.009) | **0.020** |
| Tumor encapsulation (complete versus none) | 1.547(1.127-2.125) | **0.007** |  | 1.576(1.161-2.141) | **0.004** |
| Tumor differentiation (I + II versus III + IV) | 1.499(1.056-2.128) | **0.024** |  | NA | NA |
| TNM stage (I versus II+III) | NA | NA |  | NA | NA |
| m-6-A (low versus high) | 1.462(1.052-2.031) | **0.024** |  | 1.422(1.044-1.937) | **0.025** |

Supplementary Table S4. Univariate and Multivariate Analyses of Prognostic Factors in Hepatocellular Carcinoma (cohort 2, *n*=323)

Analyses were conducted using univariate analysis or multivariate Cox proportional hazards regression. AFP, alpha-fetoprotein; GGT, gamma glutamyl transferase; HBsAg, hepatitis B surface antigen; m-6-A, N6-methyladenosine; TNM, tumor-node-metastasis. HR, hazard ratio; CI, confidential interval; NA, not adopted.

Supplementary Table S5. Correlation between FTO and Clinicopathologic Characteristics in Hepatocellular Carcinoma (cohort 2, *n* =323)

| Clinicopathological Indexes | | FTO | | *P* |
| --- | --- | --- | --- | --- |
|  |  | Low | High |  |
| Age(year) | ≤50  >50 | 80  89 | 86  68 | 0.147 |
| Sex | Female  Male | 17  152 | 29  125 | **0.026** |
| HBsAg | Negative  Positive | 23  146 | 22  132 | 0.874 |
| AFP (ng/ml) | ≤20  >20 | 49  120 | 47  107 | 0.808 |
| GGT (U/L) | ≤54  >54 | 57  112 | 74  80 | **0.009** |
| Liver cirrhosis | No  yes | 17  152 | 20  134 | 0.485 |
| Tumor size(cm) | ≤5  >5 | 76  93 | 88  66 | **0.034** |
| Tumor number | Single  Multiple | 141  28 | 135  19 | 0.344 |
| Microvascular invasion | absence  present | 77  92 | 98  56 | **0.001** |
| Tumor encapsulation | complete  none | 84  85 | 80  74 | 0.738 |
| Tumor differentiation | I+II  III+IV | 150  19 | 106  48 | **0.000** |
| TNM stage | I  II+III | 87  82 | 77  77 | 0.824 |

Fisher’s exact tests or chi-square tests for all analyses. *P* values less than 0.05 were considered statistically significant. Boldface type indicates significant values. AFP, alpha-fetoprotein; GGT, gamma glutamyl transferase; HBsAg, hepatitis B surface antigen; FTO, fat mass and obesity-associated protein; TNM, tumor-node-metastasis.
