# Supplementary figures and images for: FTO downregulation-mediated m6A modification resulting in enhanced hepatocellular carcinoma invasion
Source: Cell Biosci. 2025 May 2;15:58. doi: 10.1186/s13578-025-01395-w (PMC12049069; doi:10.1186/s13578-025-01395-w)

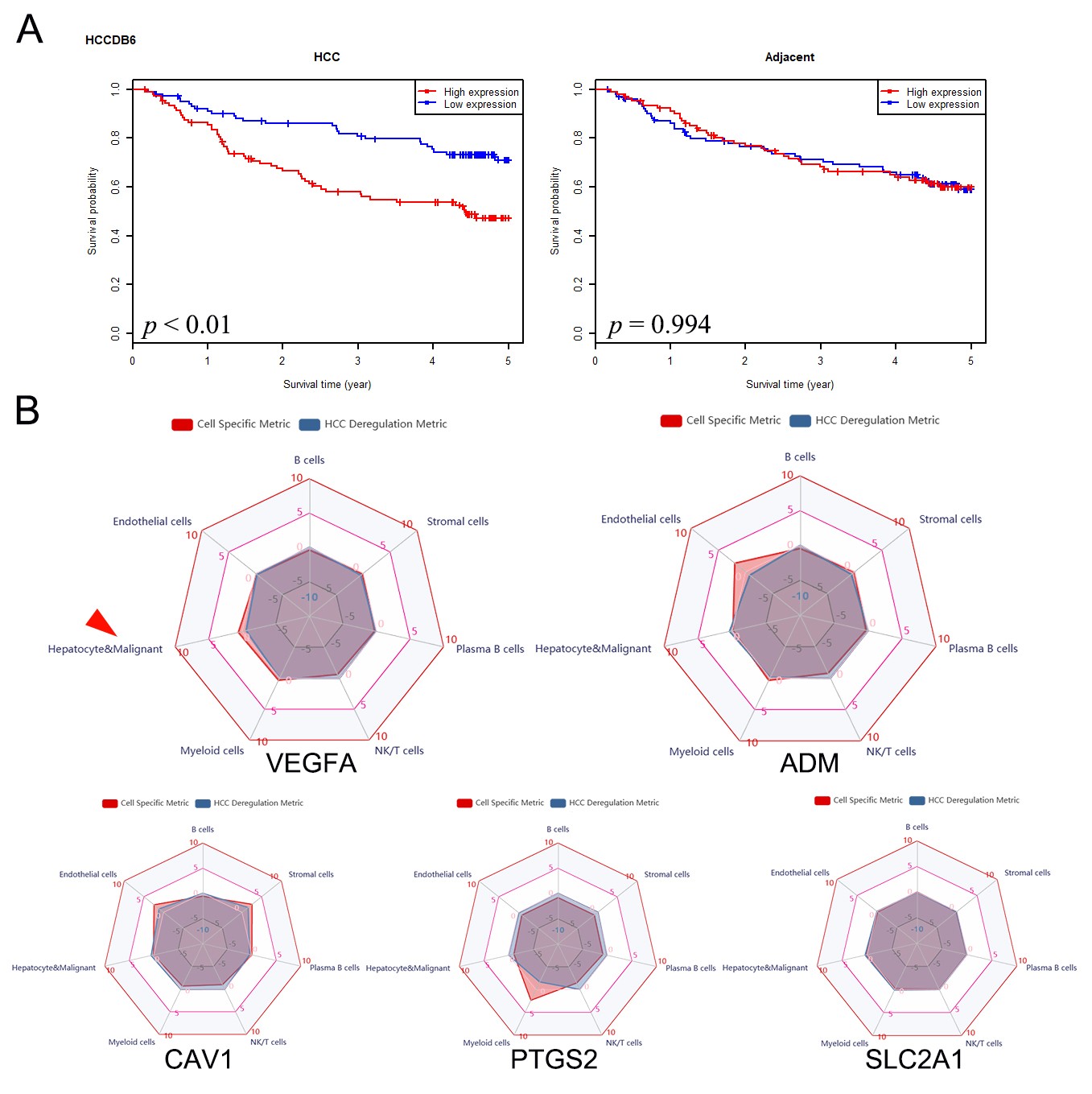

Supplement: Supplementary file 2 — Supplementary Material 2: Fig. 1. VEGFA levels in hepatocellular carcinoma are significantly correlated with the prognosis (A) and are primarily secreted by malignant cells rather than immune or stromal cells (B). [file 13578_2025_1395_MOESM2_ESM.jpg]

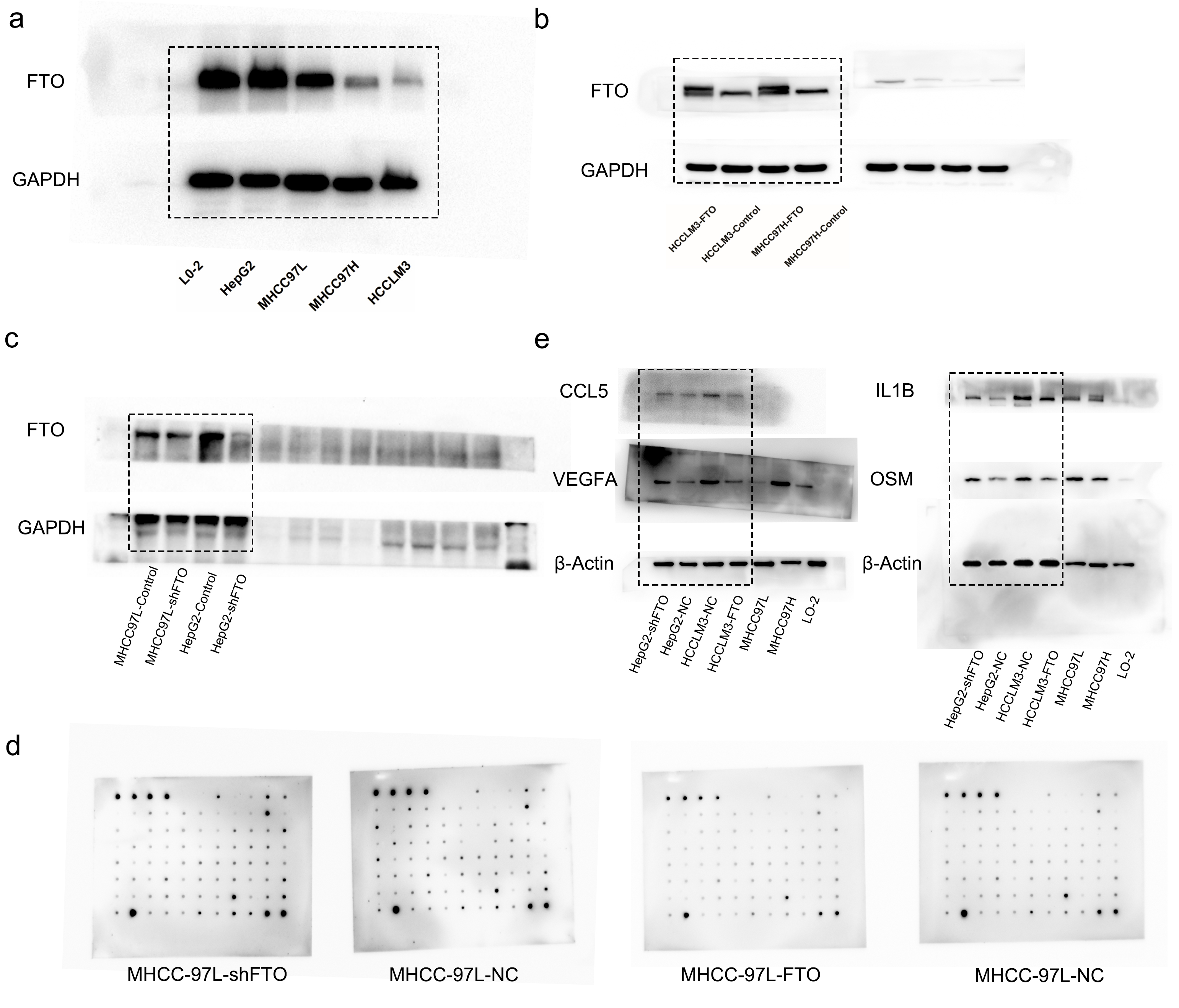

Supplement: Supplementary file 3 — Supplementary Material 3 [file 13578_2025_1395_MOESM3_ESM.jpg]
